# A global challenge and a legal requirement: new findings can help the Welsh response and put sustainable development into practice

**DOI:** 10.1093/eurpub/ckaa029

**Published:** 2020-05-11

**Authors:** Cathy Weatherup, Sumina Azam

**Affiliations:** Public Health Wales, 2 Capital Quarter, Tyndall Street, Cardiff CF10 4BZ, UK

## Abstract

Sustainable development legislation in Wales requires us to think and act differently to contribute to seven well being goals, namely; prosperity, resilience, health, equity, cohesive communities, globally responsible and a vibrant culture and thriving welsh language, now and over the longer term. Findings from a Literature Review, commissioned by the Health and Sustainability Hub, Public Health Wales, identify approaches and recommendations to help implement the legislation; aimed at people, policies and practice.

Legislation can be one of the most powerful tools available to direct long-term policy goals. It can be an effective lever for influencing change in society and provides a platform for directing action for the benefit of whole populations. In Wales, sustainable development (SD) legislation the ‘Well-being of Future Generations (Wales) Act’ places duties on national and local public services, no matter what their specific responsibilities, to maximize their contribution to improving the economic, social, environmental and cultural well-being of the country.^1^

Public bodies including Welsh Government have set and now work towards well-being objectives that demonstrate their contribution to seven statutory well-being goals. The goals serve as a translation of the range of Sustainable Development Goals into the Welsh context and include a Wales that is globally responsible, prosperous, resilient, healthier, more equal with cohesive communities and has a vibrant culture and thriving Welsh Language.

National reporting requirements are in place and include the publication of a ‘Future Trends Report’, which lists the major challenges to be addressed in Wales,^2^ and an annual ‘Well-being of Wales’ report, which assesses progress towards 46 national indicators.^3^

Accountability and scrutiny mechanisms are built into the legislation with a remit for the Auditor General for Wales to examine the extent to which public bodies have acted in accordance with the SD principle. His initial report has outlined early progress^4^ and now formal examinations have begun in each of the 44 public bodies. These examinations allow for in depth analysis and discussion with staff at all levels to assess how the organization is applying the SD principle and the five ways of working, how the organization has changed what it is doing and how the organization involves and works with its citizens and stakeholders to deliver its well-being duty.

Similarly, the establishment of a Future Generations Commissioner, with a unique role to challenge, advocate and support, has provided a view of progress through her annual report^5^ and on-going commentary on the public service response to a variety of issues such as climate change:

We have seen the pace of progress to tackling climate change gather speed here in Wales; from Welsh councils voting to divest from fossil fuels to local people taking direct action to highlight the serious threat to our health and planet as we experience some of the hottest days of the year so far across Europe. The rewriting of Planning Policy Wales in alignment with our approach to sustainable development, the declaration of a climate emergency, the commitment to 100% emissions reduction by 2050, the new focus on the foundational economy and the adoption of a definition of prevention across Government to track spending priorities.

    Sophie Howe, Future Generations Commissioner^6^

The Future Generations Commissioner has identified 10 areas for investment where action needs to be prioritized and scaled up to meet the ‘climate emergency’ challenge, including the need for investment in sustainable transport, low carbon homes and buildings, renewable energy and nature-based solutions.

## Implementing sustainable development

An important aspect of the Welsh legislation is that it defines SD as a way of doing things rather than an end in itself. It requires public bodies to use SD principles, or the five ways of working, to shape what they do, how they do it and how it is communicated. As early reports indicate,^4^^,^^5^ this still presents a challenge and it is the reason why the Health and Sustainability Hub, set up by Public Health Wales to support the implementation of the Act, commissioned a literature review on how to embed SD principles. The review identifies the approaches and methods, support materials and examples. To assist implementation, it provides five recommendations for public services to act upon, including: a recognition that the ways of working are mutually reinforcing; that interventions should be targeted at individual, team, organization and government system levels; and that all action should be visible and integrated so that we facilitate the ‘virtuous circle’ of motivation at all levels of the system and between people, policies and practice in order to secure and sustain change.

**Figure:**
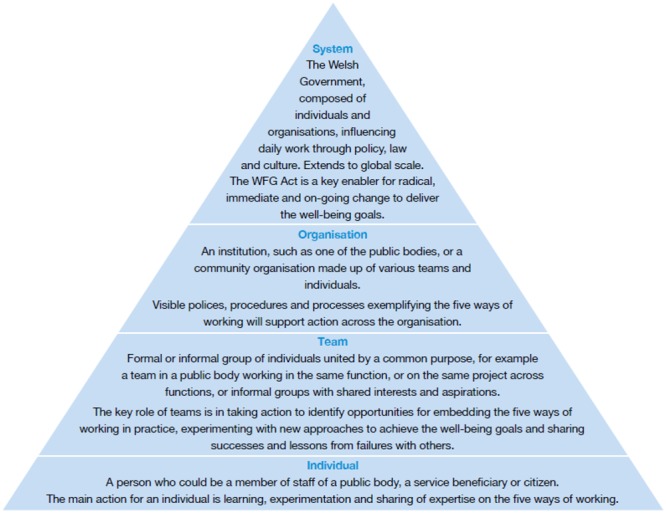
One of five recommendations on how to implement sustainable development in Wales

**Figure:**
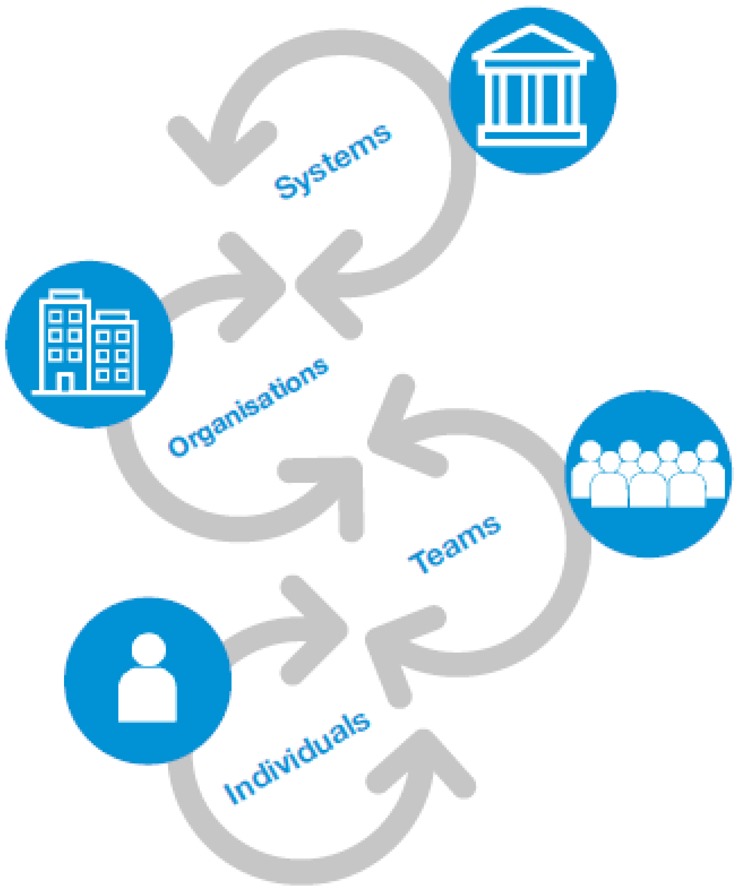
Ensure tailored support for the levels at which the five ways of working are to be implemented. There are different levels at which sustainable development can be actioned within existing structures and each supports the next as shown in the two figures At the foundation, **individuals** experience change in different ways and acknowledging and supporting staff well-being is essential to empowering individuals as change agents.At the **team** level, providing team learning and development opportunities, encouraging wide discussion within teams and permission to experiment enables visible practice of the five ways of working.At the **organisational** level, public bodies in Wales have already progressed to embed the WFG Act in polices and organisational objectives which are monitored.At the **systems** level, the WFG Act itself represents a system level enabler for radical transformation.

Furthermore, the literature review provides favourable comparisons between the Welsh and other legal frameworks that have been used to affect change, with the positive analysis indicating that the Welsh approach could make a significant and long lasting positive difference to the people living in Wales, with the potential to set an example globally.^7^

While serving the Welsh context, the findings of the review can act as a go-to document for any organization seeking to implement the globally agreed Sustainable Development Goals. As such, the review has international relevance and demonstrates how Wales, as the only country in the world to legislate for the needs of future generations, can be seen as leading the way on embedding SD into all aspects of government policy, and regional and local action.

## Conclusion

This report builds on an earlier RHN publication^8^ that provided detail of the legislation and the enabling policy, legislative and institutional context in Wales and highlighted themes such as long-standing political commitment, public support through a year-long national conversation with people and communities about what they want to leave behind for their children and grandchildren, as well as a legislative framework which requires leadership and action enacted at all levels.

Through this case study, we seek to show how SD is being translated into practice in Wales. In 2020, two reports will provide independent views on whether the Welsh SD legislation is delivering against its ambition. The first legally required Future Generations Report for Wales will be prepared and published by the Future Generations Commissioner and the Auditor General will report on his examinations to the National Assembly for Wales on progress.
